# 1,3-Bis(4-*tert*-butyl­phen­yl)-4-nitro­butan-1-one

**DOI:** 10.1107/S1600536811018277

**Published:** 2011-05-20

**Authors:** Dong-Yin Ren, Lu Shi, Qin Zhang, Yi Xu, Hong-Jun Zhu

**Affiliations:** a Department of Applied Chemistry, College of Science, Nanjing University of Technology, Nanjing 210009, People’s Republic of China

## Abstract

In the crystal structure of the title compound, C_24_H_31_NO_3_, mol­ecules are connected *via* C—H⋯O inter­molecular hydrogen bonds, forming dimers. The benzene rings are oriented at a dihedral angle of 29.8 (1)°.

## Related literature

For applications of the title compound, see: Gorman *et al.* (2004 ▶). For a related structure, see: Hall *et al.* (2005 ▶). For the synthesis of the title compound, see: Liang *et al.* (2006 ▶). For bond-length data, see: Allen *et al.* (1987 ▶).
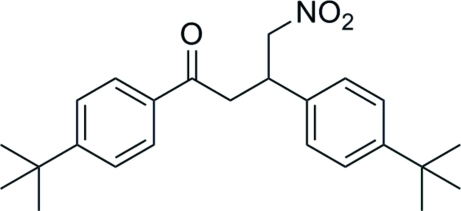


## Experimental

### 

#### Crystal data

C_24_H_31_NO_3_
*M* *_r_* = 381.50
Orthorhombic, 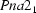

*a* = 20.440 (4) Å
*b* = 17.500 (4) Å
*c* = 6.1630 (12) Å
*V* = 2204.5 (8) Å^3^
*Z* = 4
Mo *K*α radiation
μ = 0.08 mm^−1^
*T* = 293 K
0.20 × 0.20 × 0.10 mm

#### Data collection

Enraf–Nonius CAD-4 diffractometer
Absorption correction: ψ scan (North *et al.*, 1968 ▶) *T*_min_ = 0.985, *T*_max_ = 0.993
8662 measured reflections
2247 independent reflections
1527 reflections with *I* > 2σ(*I*)
*R*_int_ = 0.096
3 standard reflections every 200 reflections  intensity decay: 1%

#### Refinement

*R*[*F* ^2^ > 2σ(*F* ^2^)] = 0.062
*wR*(*F* ^2^) = 0.181
*S* = 1.00
2247 reflections
247 parameters
1 restraint
H-atom parameters constrained
Δρ_max_ = 0.35 e Å^−3^
Δρ_min_ = −0.32 e Å^−3^

### 

Data collection: *CAD-4 Software* (Enraf-Nonius, 1985 ▶); cell refinement: *CAD-4 Software*; data reduction: *XCAD4* (Harms & Wocadlo, 1995 ▶); program(s) used to solve structure: *SHELXS97* (Sheldrick, 2008 ▶); program(s) used to refine structure: *SHELXL97* (Sheldrick, 2008 ▶); molecular graphics: *SHELXTL* (Sheldrick, 2008 ▶); software used to prepare material for publication: *SHELXTL*.

## Supplementary Material

Crystal structure: contains datablocks I, global. DOI: 10.1107/S1600536811018277/bq2291sup1.cif

Structure factors: contains datablocks I. DOI: 10.1107/S1600536811018277/bq2291Isup2.hkl

Supplementary material file. DOI: 10.1107/S1600536811018277/bq2291Isup3.cml

Additional supplementary materials:  crystallographic information; 3D view; checkCIF report

## Figures and Tables

**Table 1:** Hydrogen-bond geometry (Å, °)

*D*—H⋯*A*	*D*—H	H⋯*A*	*D*⋯*A*	*D*—H⋯*A*
C12—H12*A*⋯O1^i^	0.97	2.52	3.072 (5)	116

Symmetry code: (i) 
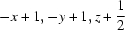
.

**Fig. 1.**
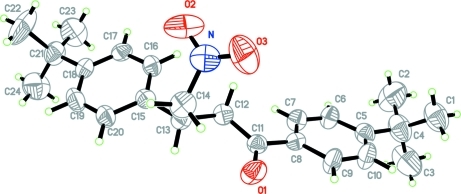
The molecular structure of (I), with the atom-numbering scheme. Displacement ellipsoids are drawn at the 50% probability level.

**Fig. 2.**
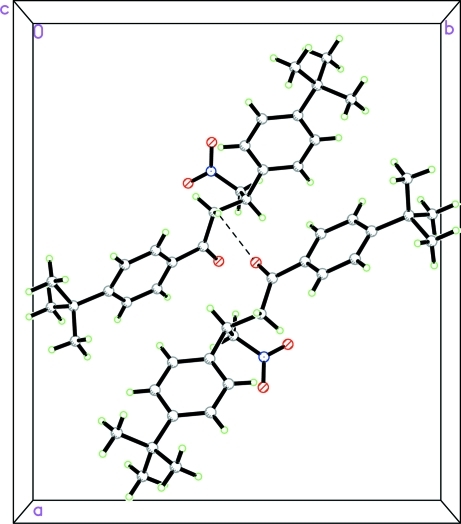
A packing diagram of (I). Hydrogen bonds are shown as dashed lines.

## supplementary crystallographic information

### Comment

The tittle compound, 1,3-bis(4-*tert*-butylphenyl)-4-nitrobutan-1-one is an important intermediate (Gorman *et al.*, 2004) for the synthesis of 2,4-bis(4-*tert*-butylphenyl)-4-oxobutanal, which can be used to synthesis 2,4-bis(4-*tert*-butylphenyl)-1*H*-pyrrole(Hall *et al.*, 2005). We report here the crystal structure of the title compound, (I).

The molecular structure of (I) is shown in Fig. 1. The dihedral angle between the rings (C5—C10) and (C15—C20) is 29.8 (1)°. The bond lengths and angles are within normal ranges (Allen *et al.*, 1987).

The molecules of (I) were connected together *via* C—H···O intermolecular hydrogen bonds to form molecular dimers (Table 1. and Fig. 2.).

### Experimental

The title compound, (I) was prepared by the method of Michael reaction reported in literature (Liang *et al.*, 2006). The crystals were obtained by dissolving (I) (0.2 g, 0.52 mmol) in ethanol (25 ml) and evaporating the solvent slowly at room temperature for about 5 d.

### Refinement

H atoms were positioned geometrically and refined as riding groups, with C—H = 0.93, 0.96, 0.97 and 0.98 Å for aromatic, methyl, methylene and methine H, respectively, and constrained to ride on their parent atoms, with *U*_iso_(H) = *xU*_eq_(C), where *x* = 1.2 for aromatic H, and *x* = 1.5 for other H.

### Crystal data

**Table:**

C_24_H_31_NO_3_	*F*(000) = 824
*M**_r_* = 381.50	*D*_x_ = 1.149 Mg m^−^^3^
Orthorhombic, *P**n**a*2_1_	Mo *K*α radiation, λ = 0.71073 Å
Hall symbol: P 2c -2n	Cell parameters from 25 reflections
*a* = 20.440 (4) Å	θ = 9–13°
*b* = 17.500 (4) Å	µ = 0.08 mm^−^^1^
*c* = 6.1630 (12) Å	*T* = 293 K
*V* = 2204.5 (8) Å^3^	Block, colourless
*Z* = 4	0.20 × 0.20 × 0.10 mm

### Data collection

**Table:**

Enraf–Nonius CAD-4 diffractometer	1527 reflections with *I* > 2σ(*I*)
Radiation source: fine-focus sealed tube	*R*_int_ = 0.096
graphite	θ_max_ = 25.4°, θ_min_ = 1.5°
ω/2θ scans	*h* = −24→24
Absorption correction: ψ scan (North *et al.*, 1968)	*k* = −21→21
*T*_min_ = 0.985, *T*_max_ = 0.993	*l* = −7→0
8662 measured reflections	3 standard reflections every 200 reflections
2247 independent reflections	intensity decay: 1%

### Refinement

**Table:**

Refinement on *F*^2^	Primary atom site location: structure-invariant direct methods
Least-squares matrix: full	Secondary atom site location: difference Fourier map
*R*[*F*^2^ > 2σ(*F*^2^)] = 0.062	Hydrogen site location: inferred from neighbouring sites
*wR*(*F*^2^) = 0.181	H-atom parameters constrained
*S* = 1.00	*w* = 1/[σ^2^(*F*_o_^2^) + (0.1*P*)^2^ + 0.3*P*] where *P* = (*F*_o_^2^ + 2*F*_c_^2^)/3
2247 reflections	(Δ/σ)_max_ < 0.001
247 parameters	Δρ_max_ = 0.35 e Å^−^^3^
1 restraint	Δρ_min_ = −0.32 e Å^−^^3^

### Special details

**Table:**

Geometry. All e.s.d.'s (except the e.s.d. in the dihedral angle between two l.s. planes) are estimated using the full covariance matrix. The cell e.s.d.'s are taken into account individually in the estimation of e.s.d.'s in distances, angles and torsion angles; correlations between e.s.d.'s in cell parameters are only used when they are defined by crystal symmetry. An approximate (isotropic) treatment of cell e.s.d.'s is used for estimating e.s.d.'s involving l.s. planes.
Refinement. Refinement of *F*^2^ against ALL reflections. The weighted *R*-factor *wR* and goodness of fit *S* are based on *F*^2^, conventional *R*-factors *R* are based on *F*, with *F* set to zero for negative *F*^2^. The threshold expression of *F*^2^ > σ(*F*^2^) is used only for calculating *R*-factors(gt) *etc*. and is not relevant to the choice of reflections for refinement. *R*-factors based on *F*^2^ are statistically about twice as large as those based on *F*, and *R*- factors based on ALL data will be even larger.

### Fractional atomic coordinates and isotropic or equivalent isotropic displacement parameters (Å^2^)

**Table:**

	*x*	*y*	*z*	*U*_iso_*/*U*_eq_	
N	0.6807 (2)	0.5602 (3)	0.0686 (7)	0.0600 (11)	
O1	0.50125 (15)	0.54161 (17)	0.2287 (6)	0.0631 (10)	
C1	0.4134 (3)	0.9145 (3)	0.5244 (13)	0.0861 (15)	
H1A	0.3856	0.8949	0.4118	0.129*	
H1B	0.4564	0.9235	0.4669	0.129*	
H1C	0.3957	0.9615	0.5788	0.129*	
O2	0.73656 (19)	0.5582 (3)	0.1435 (8)	0.0888 (14)	
C2	0.4638 (4)	0.8985 (4)	0.8758 (18)	0.128 (4)	
H2A	0.4453	0.9465	0.9193	0.192*	
H2B	0.5063	0.9070	0.8136	0.192*	
H2C	0.4679	0.8658	1.0001	0.192*	
O3	0.6580 (2)	0.6145 (2)	−0.0204 (9)	0.0976 (16)	
C3	0.3532 (4)	0.8505 (4)	0.813 (2)	0.141 (4)	
H3A	0.3221	0.8277	0.7162	0.211*	
H3B	0.3377	0.8999	0.8576	0.211*	
H3C	0.3585	0.8185	0.9385	0.211*	
C4	0.4174 (3)	0.8589 (3)	0.7002 (13)	0.0861 (15)	
C5	0.4448 (2)	0.7832 (3)	0.6253 (9)	0.0524 (12)	
C6	0.4948 (2)	0.7455 (3)	0.7359 (9)	0.0549 (12)	
H6A	0.5103	0.7660	0.8652	0.066*	
C7	0.5216 (2)	0.6792 (2)	0.6595 (8)	0.0505 (12)	
H7A	0.5552	0.6560	0.7369	0.061*	
C8	0.49945 (19)	0.6457 (2)	0.4682 (8)	0.0405 (10)	
C9	0.4479 (2)	0.6811 (3)	0.3611 (10)	0.0569 (13)	
H9A	0.4306	0.6594	0.2358	0.068*	
C10	0.4224 (3)	0.7479 (3)	0.4400 (11)	0.0654 (15)	
H10A	0.3882	0.7707	0.3644	0.078*	
C11	0.5300 (2)	0.5762 (2)	0.3703 (8)	0.0416 (10)	
C12	0.5950 (2)	0.5512 (2)	0.4511 (8)	0.0418 (10)	
H12A	0.5919	0.5420	0.6059	0.050*	
H12B	0.6260	0.5925	0.4295	0.050*	
C13	0.6218 (2)	0.4789 (2)	0.3414 (8)	0.0427 (11)	
H13A	0.5858	0.4420	0.3412	0.051*	
C14	0.6395 (2)	0.4918 (3)	0.1035 (9)	0.0530 (12)	
H14A	0.6626	0.4472	0.0497	0.064*	
H14B	0.5996	0.4971	0.0199	0.064*	
C15	0.6768 (2)	0.4417 (2)	0.4692 (8)	0.0435 (10)	
C16	0.7202 (2)	0.4823 (2)	0.5969 (9)	0.0453 (11)	
H16A	0.7172	0.5352	0.6026	0.054*	
C17	0.76808 (19)	0.4452 (2)	0.7167 (9)	0.0460 (11)	
H17A	0.7970	0.4742	0.7989	0.055*	
C18	0.7743 (2)	0.3668 (2)	0.7183 (9)	0.0466 (11)	
C19	0.7315 (2)	0.3266 (2)	0.5847 (11)	0.0605 (14)	
H19A	0.7358	0.2739	0.5732	0.073*	
C20	0.6833 (2)	0.3630 (2)	0.4697 (10)	0.0538 (12)	
H20A	0.6540	0.3338	0.3893	0.065*	
C21	0.8267 (3)	0.3263 (3)	0.8525 (11)	0.0643 (14)	
C22	0.8825 (3)	0.3032 (5)	0.6997 (15)	0.119 (3)	
H22A	0.9157	0.2770	0.7807	0.179*	
H22B	0.9008	0.3481	0.6342	0.179*	
H22C	0.8658	0.2700	0.5886	0.179*	
C23	0.8533 (4)	0.3767 (4)	1.0330 (14)	0.103 (3)	
H23A	0.8183	0.3911	1.1285	0.154*	
H23B	0.8725	0.4218	0.9713	0.154*	
H23C	0.8859	0.3491	1.1133	0.154*	
C24	0.7971 (3)	0.2562 (4)	0.9646 (15)	0.099 (2)	
H24A	0.7630	0.2722	1.0617	0.148*	
H24B	0.8305	0.2300	1.0450	0.148*	
H24C	0.7791	0.2224	0.8572	0.148*	

### Atomic displacement parameters (Å^2^)

**Table:**

	*U*^11^	*U*^22^	*U*^33^	*U*^12^	*U*^13^	*U*^23^
N	0.069 (3)	0.076 (3)	0.035 (2)	−0.006 (2)	0.010 (2)	0.002 (2)
O1	0.0523 (18)	0.0704 (19)	0.066 (2)	0.0060 (16)	−0.0210 (19)	−0.028 (2)
C1	0.108 (3)	0.061 (2)	0.089 (4)	0.013 (3)	0.026 (3)	0.002 (2)
O2	0.066 (2)	0.131 (3)	0.069 (3)	−0.036 (2)	0.002 (2)	0.011 (3)
C2	0.106 (5)	0.105 (5)	0.174 (10)	0.027 (4)	−0.021 (7)	−0.067 (7)
O3	0.137 (4)	0.071 (3)	0.086 (3)	0.005 (2)	−0.014 (4)	0.026 (3)
C3	0.124 (6)	0.083 (4)	0.214 (12)	0.017 (4)	0.097 (8)	0.004 (6)
C4	0.108 (3)	0.061 (2)	0.089 (4)	0.013 (3)	0.026 (3)	0.002 (2)
C5	0.055 (3)	0.046 (2)	0.056 (3)	0.002 (2)	0.008 (3)	−0.003 (3)
C6	0.059 (3)	0.058 (3)	0.047 (3)	0.014 (2)	−0.005 (3)	−0.008 (3)
C7	0.054 (3)	0.056 (3)	0.042 (3)	0.005 (2)	−0.006 (2)	−0.005 (2)
C8	0.036 (2)	0.045 (2)	0.040 (2)	−0.0024 (17)	−0.001 (2)	0.000 (2)
C9	0.055 (3)	0.056 (3)	0.060 (3)	0.020 (2)	−0.017 (3)	−0.011 (3)
C10	0.063 (3)	0.062 (3)	0.071 (4)	0.023 (3)	−0.016 (3)	0.000 (3)
C11	0.041 (2)	0.041 (2)	0.043 (3)	−0.0027 (18)	0.003 (2)	0.000 (2)
C12	0.047 (2)	0.046 (2)	0.032 (2)	0.0030 (18)	0.012 (2)	0.001 (2)
C13	0.041 (2)	0.046 (2)	0.041 (3)	0.0016 (19)	−0.003 (2)	−0.002 (2)
C14	0.057 (3)	0.063 (3)	0.039 (3)	0.001 (2)	−0.001 (3)	−0.005 (2)
C15	0.046 (2)	0.042 (2)	0.043 (3)	−0.0025 (18)	0.011 (2)	−0.002 (2)
C16	0.054 (2)	0.039 (2)	0.042 (3)	0.0018 (19)	0.002 (2)	−0.003 (2)
C17	0.036 (2)	0.056 (3)	0.045 (3)	−0.0036 (18)	−0.008 (2)	−0.005 (3)
C18	0.041 (2)	0.050 (2)	0.049 (3)	0.0076 (19)	−0.001 (2)	0.001 (3)
C19	0.070 (3)	0.037 (2)	0.075 (4)	0.003 (2)	−0.004 (3)	−0.008 (3)
C20	0.053 (3)	0.041 (2)	0.068 (3)	0.002 (2)	−0.008 (3)	−0.013 (3)
C21	0.062 (3)	0.066 (3)	0.065 (3)	0.014 (3)	−0.005 (3)	0.014 (3)
C22	0.081 (4)	0.160 (7)	0.117 (7)	0.070 (4)	0.024 (5)	0.044 (6)
C23	0.108 (5)	0.096 (4)	0.104 (6)	0.003 (4)	−0.055 (5)	0.004 (4)
C24	0.109 (5)	0.084 (4)	0.105 (6)	−0.001 (4)	−0.014 (5)	0.042 (5)

### Geometric parameters (Å, °)

**Table:**

N—O3	1.192 (5)	C12—H12A	0.9700
N—O2	1.231 (5)	C12—H12B	0.9700
N—C14	1.479 (6)	C13—C15	1.520 (6)
O1—C11	1.214 (5)	C13—C14	1.527 (7)
C1—C4	1.457 (9)	C13—H13A	0.9800
C1—H1A	0.9600	C14—H14A	0.9700
C1—H1B	0.9600	C14—H14B	0.9700
C1—H1C	0.9600	C15—C16	1.381 (6)
C2—C4	1.597 (11)	C15—C20	1.384 (6)
C2—H2A	0.9600	C16—C17	1.388 (6)
C2—H2B	0.9600	C16—H16A	0.9300
C2—H2C	0.9600	C17—C18	1.377 (6)
C3—C4	1.493 (9)	C17—H17A	0.9300
C3—H3A	0.9600	C18—C19	1.392 (7)
C3—H3B	0.9600	C18—C21	1.528 (7)
C3—H3C	0.9600	C19—C20	1.371 (7)
C4—C5	1.511 (7)	C19—H19A	0.9300
C5—C10	1.377 (8)	C20—H20A	0.9300
C5—C6	1.394 (7)	C21—C23	1.520 (9)
C6—C7	1.367 (6)	C21—C24	1.533 (8)
C6—H6A	0.9300	C21—C22	1.533 (9)
C7—C8	1.392 (6)	C22—H22A	0.9600
C7—H7A	0.9300	C22—H22B	0.9600
C8—C9	1.390 (6)	C22—H22C	0.9600
C8—C11	1.494 (6)	C23—H23A	0.9600
C9—C10	1.369 (6)	C23—H23B	0.9600
C9—H9A	0.9300	C23—H23C	0.9600
C10—H10A	0.9300	C24—H24A	0.9600
C11—C12	1.485 (6)	C24—H24B	0.9600
C12—C13	1.536 (6)	C24—H24C	0.9600
			
O3—N—O2	123.9 (5)	C15—C13—C14	112.7 (4)
O3—N—C14	119.3 (5)	C15—C13—C12	112.9 (4)
O2—N—C14	116.8 (5)	C14—C13—C12	112.7 (4)
C4—C1—H1A	109.5	C15—C13—H13A	105.9
C4—C1—H1B	109.5	C14—C13—H13A	105.9
H1A—C1—H1B	109.5	C12—C13—H13A	105.9
C4—C1—H1C	109.5	N—C14—C13	113.2 (4)
H1A—C1—H1C	109.5	N—C14—H14A	108.9
H1B—C1—H1C	109.5	C13—C14—H14A	108.9
C4—C2—H2A	109.5	N—C14—H14B	108.9
C4—C2—H2B	109.5	C13—C14—H14B	108.9
H2A—C2—H2B	109.5	H14A—C14—H14B	107.8
C4—C2—H2C	109.5	C16—C15—C20	116.6 (4)
H2A—C2—H2C	109.5	C16—C15—C13	123.4 (4)
H2B—C2—H2C	109.5	C20—C15—C13	119.9 (4)
C4—C3—H3A	109.5	C15—C16—C17	121.1 (4)
C4—C3—H3B	109.5	C15—C16—H16A	119.5
H3A—C3—H3B	109.5	C17—C16—H16A	119.5
C4—C3—H3C	109.5	C18—C17—C16	122.3 (4)
H3A—C3—H3C	109.5	C18—C17—H17A	118.8
H3B—C3—H3C	109.5	C16—C17—H17A	118.8
C1—C4—C3	111.3 (6)	C17—C18—C19	116.2 (4)
C1—C4—C5	112.2 (6)	C17—C18—C21	122.1 (4)
C3—C4—C5	112.4 (5)	C19—C18—C21	121.7 (4)
C1—C4—C2	104.4 (6)	C20—C19—C18	121.5 (4)
C3—C4—C2	104.4 (7)	C20—C19—H19A	119.2
C5—C4—C2	111.5 (6)	C18—C19—H19A	119.2
C10—C5—C6	115.9 (4)	C19—C20—C15	122.2 (5)
C10—C5—C4	121.6 (5)	C19—C20—H20A	118.9
C6—C5—C4	122.5 (5)	C15—C20—H20A	118.9
C7—C6—C5	121.8 (5)	C23—C21—C18	112.2 (4)
C7—C6—H6A	119.1	C23—C21—C24	106.0 (6)
C5—C6—H6A	119.1	C18—C21—C24	109.8 (5)
C6—C7—C8	121.2 (5)	C23—C21—C22	109.7 (6)
C6—C7—H7A	119.4	C18—C21—C22	108.1 (5)
C8—C7—H7A	119.4	C24—C21—C22	111.1 (5)
C9—C8—C7	117.4 (4)	C21—C22—H22A	109.5
C9—C8—C11	119.2 (4)	C21—C22—H22B	109.5
C7—C8—C11	123.3 (4)	H22A—C22—H22B	109.5
C10—C9—C8	120.1 (5)	C21—C22—H22C	109.5
C10—C9—H9A	120.0	H22A—C22—H22C	109.5
C8—C9—H9A	120.0	H22B—C22—H22C	109.5
C9—C10—C5	123.4 (5)	C21—C23—H23A	109.5
C9—C10—H10A	118.3	C21—C23—H23B	109.5
C5—C10—H10A	118.3	H23A—C23—H23B	109.5
O1—C11—C12	121.8 (4)	C21—C23—H23C	109.5
O1—C11—C8	119.6 (4)	H23A—C23—H23C	109.5
C12—C11—C8	118.6 (4)	H23B—C23—H23C	109.5
C11—C12—C13	114.5 (4)	C21—C24—H24A	109.5
C11—C12—H12A	108.6	C21—C24—H24B	109.5
C13—C12—H12A	108.6	H24A—C24—H24B	109.5
C11—C12—H12B	108.6	C21—C24—H24C	109.5
C13—C12—H12B	108.6	H24A—C24—H24C	109.5
H12A—C12—H12B	107.6	H24B—C24—H24C	109.5
			
C1—C4—C5—C10	−47.4 (8)	O3—N—C14—C13	−110.0 (5)
C3—C4—C5—C10	79.0 (8)	O2—N—C14—C13	66.9 (6)
C2—C4—C5—C10	−164.1 (6)	C15—C13—C14—N	−79.7 (5)
C1—C4—C5—C6	131.4 (6)	C12—C13—C14—N	49.5 (5)
C3—C4—C5—C6	−102.2 (8)	C14—C13—C15—C16	98.0 (5)
C2—C4—C5—C6	14.8 (8)	C12—C13—C15—C16	−31.1 (6)
C10—C5—C6—C7	2.6 (7)	C14—C13—C15—C20	−85.2 (6)
C4—C5—C6—C7	−176.3 (5)	C12—C13—C15—C20	145.7 (5)
C5—C6—C7—C8	−0.8 (7)	C20—C15—C16—C17	0.7 (7)
C6—C7—C8—C9	−1.8 (6)	C13—C15—C16—C17	177.6 (4)
C6—C7—C8—C11	175.8 (4)	C15—C16—C17—C18	−1.2 (8)
C7—C8—C9—C10	2.4 (7)	C16—C17—C18—C19	2.8 (7)
C11—C8—C9—C10	−175.2 (5)	C16—C17—C18—C21	−179.3 (5)
C8—C9—C10—C5	−0.6 (8)	C17—C18—C19—C20	−4.1 (8)
C6—C5—C10—C9	−1.9 (8)	C21—C18—C19—C20	178.0 (5)
C4—C5—C10—C9	177.0 (6)	C18—C19—C20—C15	4.0 (9)
C9—C8—C11—O1	−16.6 (6)	C16—C15—C20—C19	−2.1 (8)
C7—C8—C11—O1	165.9 (4)	C13—C15—C20—C19	−179.1 (5)
C9—C8—C11—C12	163.8 (4)	C17—C18—C21—C23	19.4 (8)
C7—C8—C11—C12	−13.7 (6)	C19—C18—C21—C23	−162.8 (6)
O1—C11—C12—C13	−0.1 (6)	C17—C18—C21—C24	137.1 (6)
C8—C11—C12—C13	179.5 (4)	C19—C18—C21—C24	−45.2 (8)
C11—C12—C13—C15	−162.8 (4)	C17—C18—C21—C22	−101.6 (7)
C11—C12—C13—C14	68.1 (5)	C19—C18—C21—C22	76.2 (7)

### Hydrogen-bond geometry (Å, °)

**Table:**

*D*—H···*A*	*D*—H	H···*A*	*D*···*A*	*D*—H···*A*
C12—H12A···O1^i^	0.97	2.52	3.072 (5)	116

Symmetry codes: (i) −*x*+1, −*y*+1, *z*+1/2.
